# Increased abundance of *Sutterella spp.* and *Ruminococcus torques* in feces of children with autism spectrum disorder

**DOI:** 10.1186/2040-2392-4-42

**Published:** 2013-11-04

**Authors:** Lv Wang, Claus T Christophersen, Michael J Sorich, Jacobus P Gerber, Manya T Angley, Michael A Conlon

**Affiliations:** 1 Sansom Institute for Health Research, University of South Australia, GPO Box 2471, Adelaide, South Australia 5001, Australia; 2 Preventative Health National Research Flagship, CSIRO Animal, Food and Health Sciences, Gate 13, Kintore Avenue, Adelaide, South Australia 5001, Australia

**Keywords:** Autism spectrum disorder, Gut, Feces, Microbiota, *Sutterella*

## Abstract

**Background:**

A recent report indicated that numbers of *Sutterella spp.* are elevated in gastrointestinal biopsies taken from children with autism spectrum disorder (ASD). We have recently reported changes in the numbers of some bacteria within the stool of ASD children, and now examine whether numbers of *Sutterella spp.* and some other mucosa-associated bacteria linked with gastrointestinal disease (*Ruminococcus gnavus* and *Ruminococcus torques*) are also altered in the stool of these children.

**Findings:**

We show that numbers of *Sutterella spp.* are elevated in feces of ASD children relative to controls, and that numbers of *R. torques* are higher in the children with ASD with a reported functional gastrointestinal disorder than those without such a disorder.

**Conclusions:**

We show further evidence of changes in the gut microbiota of children with ASD and confirm that the abundance of *Sutterella spp.* is altered in stool.

## Findings

Autism spectrum disorder (ASD) is a neurodevelopmental disorder where there is evidence of gastrointestinal (GI) disturbance in many affected individuals. Several studies have demonstrated an altered GI microbiota in children with ASD compared with controls [1-4]. Recently, Williams *et al.*[5] reported a significantly higher prevalence of *Sutterella spp.* in biopsies taken from the GI tract of ASD children with GI disturbance compared to controls with GI disturbance. Bacteria of the genus *Sutterella* have been identified in canine and human feces [6,7]. *Sutterella wadsworthensis* is noteworthy as it has been associated with some GI infections in humans [8]. In one study using culture-based techniques, less than 1% of individuals had detectable fecal levels of *S. wadsworthensis*, even among individuals with bowel disorders [9]. However, in another study, where it was shown that the abundance of *S. wadsworthensis* in colonic biopsies of adults with inflammatory bowel disease (IBD) was not different from controls, *Sutterella* appeared to be more prevalent in the human gut than previously reported [10].

A previous study by our group used quantitative real-time PCR (QPCR) to compare the abundance of a range of bacteria in feces of children with ASD, their siblings and community controls, and demonstrated a low relative abundance of *Bifidobacterium spp.* and the mucolytic bacterium *Akkermansia muciniphila* in children with ASD [11]. We have carried out further QPCR analyses of samples from that study to examine whether the abundance of *Sutterella spp.* is also altered in the feces of children with ASD relative to controls. We have also examined whether fecal numbers of some other mucosa-associated bacteria, namely *Ruminococcus torques* and *Ruminococcus gnavus*, are altered in individuals with ASD. As with *A. muciniphila*, mucosal numbers of *Ruminococcus* species are altered in individuals with IBD [12].

Fecal samples were collected from 23 children with ASD (3 without siblings), 22 typically developing siblings (SIB) and 9 community controls (CON) without a family history of ASD. The study complied with National Health and Medical Research Council (Australia) guidelines relating to ethical conduct in human research. Participants’ caregivers completed a functional GI disorder (FGID) questionnaire [11]. Procedures for fecal sample collection and processing, as well as microbial DNA extraction, have been described previously [11]. QPCR was used to estimate the abundance of *Sutterella spp.*, *R. torques* and *R. gnavus* in feces using previously described primers [5,12]. Methods for QPCR, data analysis and primers for total bacteria were also as reported previously [11]. For *Sutterella*, SsoFast™ Probes Supermix was used instead of EvaGreen® Supermix and an updated version of Bio-Rad CFX Manager software (Version 2.0) was used. Statistical analyses were carried out using SPSS for Windows™ (version 17, SPSS Inc., Chicago, IL, USA). Analyses were carried out using analysis of variance (ANOVA) followed by Dunnett’s test or by Student’s *t*-test. A *P* value of less than 0.05 was considered statistically significant.

The absolute numbers (per gram of feces) and relative numbers of bacteria in feces of individuals from the ASD, SIB and CON groups are presented in Table 1. *Sutterella* was detected in the majority of samples analyzed (ASD 21/23; SIB 19/22; CON 9/9). ANOVA revealed significantly different absolute and relative numbers of *Sutterella* in ASD children compared with sibling and community controls (*P* = 0.044 and *P* = 0.05, respectively). A *post hoc* analysis showed significantly increased absolute and relative numbers of *Sutterella* in individuals with ASD compared with community controls (*P* = 0.028 and *P* = 0.03, respectively). Moreover, absolute fecal numbers of *Sutterella* tended to be higher in siblings than community controls (*P* = 0.047). *R. torques* and *R. gnavus* were detected in all fecal samples. Absolute numbers of *R. torques* were not significantly higher in ASD children than in other groups (ANOVA, *P* = 0.08) but were higher in siblings relative to community controls (*P* = 0.046). Absolute and relative numbers of *R. gnavus* did not differ significantly between groups. Significantly elevated absolute numbers of *R. torques* were evident in children with ASD whose caregivers reported them having a FGID (nine individuals) compared to those without reported FGID (14 individuals) (Student’s *t*-test, *P* = 0.008). There was no significant difference in fecal numbers of *Sutterella* or *R. gnavus* between ASD children with and without reported FGIDs (data not shown).

**Table 1:** Numbers of targeted bacteria in feces of children with ASD, their siblings and community controls

**Bacterial targets**	**Bacterial number (mean ± standard error of the mean)**		
	**Autism spectrum disorder (*n *** **= 23)**	**Siblings (*n *** **= 22)**	**Community controls (*n *** **= 9)**
**Absolute number per gram feces**			
*Sutterella spp.*	(1.1 ± 0.4) × 10^6*^	(8.7 ± 2.5) × 10^5*^	(6.7 ± 4.5) × 10^5^
*R. torques*	(3.5 ± 1.6) × 10^5^	(4.2 ± 1.5) × 10^5*^	(3.5 ± 1.4) × 10^4^
*R. gnavus*	(4.5 ± 0.7) × 10^5^	(5.1 ± 0.9) × 10^5^	(2.9 ± 0.4) × 10^5^
Total bacteria	(1.2 ± 0.2) × 10^9^	(1.4 ± 0.3) × 10^9^	(1.3 ± 0.2) × 10^9^
**Relative numbers**^**2**^			
*Sutterella spp.*	3.0 ± 0.7^*^	7.4 ± 3.5^*^	0.5 ± 0.3
*R. torques*	4.8 ± 1.8	4.8 ± 1.2	1.1 ± 0.2
*R. gnavus*	1.3 ± 0.2	1.8 ± 0.5	0.6 ± 0.2

^1^Means with an asterisk differ from those of community controls. *P* < 0.05 (one-way ANOVA followed by Dunnett’s test).

^2^Relative numbers were calculated using qBase+ [13,14].

*ASD* autism spectrum disorder.

Our results demonstrate that numbers of *Sutterella* are elevated in the feces of children with ASD relative to community controls. This confirms and builds on the findings of Williams *et al.*[5], who showed high detection rates of these bacteria in GI biopsies taken from ASD children with a GI disturbance. In our study there was no evidence of a difference in numbers of *Sutterella* between ASD children with or without caregiver-reported FGID. It is not yet evident what the consequences of an increase in fecal *Sutterella* populations means but it is possible that under specific conditions these bacteria could cause infection. Our findings add to the growing list of bacterial groups whose abundance appears to be altered in the GI tract of children with ASD. Our results also indicate that fecal samples may suffice for the detection and quantification of *Sutterella* in the human gut, including children with ASD. This contrasts with a previous estimation of detectability and abundance in human fecal samples by Engberg *et al*. [9]. Previously it has been difficult to culture and isolate anaerobic and microaerophilic bacteria, such as *Sutterella*, using traditional microbiological techniques and it is likely the QPCR method we employed was more sensitive than the culture method used by the previous study [9]. However, our detection rates of *Sutterella* in humans are comparable with detection rates in colonic biopsies from human adults [10].

In this study we also extended our analysis of mucus-degrading bacteria in feces of ASD children, having previously shown a low abundance of the mucolytic bacterium *A. muciniphila*. We measured the abundances of *R. torques* and *R. gnavus*, both of which can degrade mucus [15,16] and have been associated with GI disturbance [17]. We have shown there is a trend of increased fecal numbers of *R. torques* in ASD children. This pattern of decreased numbers of *A. muciniphila* but increased numbers of *R. torques* in feces of ASD children is congruent with the pattern observed in the mucosa of adults with IBD [12]. In the latter study it was also found that growth of *A. muciniphila* was inhibited by co-culture with MUC2, the predominant form of mucin in the large bowel, whereas this mucin augmented the growth of other bacteria such as *R. torques*. Although it is likely that the populations and activities of microbes that adhere to the mucus lining the large bowel may differ substantially from those in the feces, it is also likely that some changes in gut mucosal microbial populations are reflected in, and hence detectable in, feces. Changes in the amount of mucus produced may drive changes in mucus-degrading microbes in both the mucosa and in feces, as mucus can be incorporated into the fecal stream. Individuals with IBD or ASD may have changes in large bowel mucus production that could impact (or are a result of changes in) the mucosal barrier of the gut. Indeed, increased gut permeability has been reported in a subgroup of children with ASD [18,19]. Further studies that look at gut mucus production and turnover in children with ASD are warranted.

In summary, we show further evidence of changes in the gut microbiota of children with ASD, now demonstrating that fecal abundances of *Sutterella* and *R. torques* are altered (the latter in those reported to have FGID). The role that these particular bacteria play in ASD is not yet understood but they may simply reflect larger scale shifts in the microbial populations as a result of the condition.

## Abbreviations

ANOVA: Analysis of variance; ASD: Autism spectrum disorder; CON: Control group; FGID: Functional gastrointestinal disorder; GI: Gastrointestinal; IBD: Inflammatory bowel disease; QPCR: Quantitative real-time polymerase chain reaction; SIB: Sibling group.

## Competing interests

The authors declare that they have no competing interests.

## Authors’ contributions

CTC, JPG, LW, MAC, MJS and MTA contributed to the design and planning of the study. LW conducted the experiment and analytical methods. CTC, LW and MAC contributed to the analysis of the results, LW, MAC and MTA prepared the manuscript, and all authors read and approved the manuscript.
